# *In vitro* investigation of the antiviral activity of propolis and chitosan nanoparticles against the genotype VII Newcastle disease virus

**DOI:** 10.3389/fvets.2022.947641

**Published:** 2022-08-25

**Authors:** Noura Alkhalefa, Samy Khaliel, Abdelnaby Tahoon, Hanan Shaban, Asmaa Magouz, Hanaa Ghabban, Maha S. Lokman, Ehab Kotb Elmahallawy

**Affiliations:** ^1^Department of Virology, Faculty of Veterinary Medicine, Kafrelsheik University, Kafr El-Sheikh, Egypt; ^2^Department of Microbiology, Faculty of Veterinary Medicine, Alexandria University, Alexandria, Egypt; ^3^Animal Health Research Institute, Kafrelsheik Lab, Agriculture Research Center (ARC), Giza, Egypt; ^4^Department of Biology, Faculty of Science, Tabuk University, Tabuk, Saudi Arabia; ^5^Department of Biology, College of Science and Humanities in Al-Kharj, Prince Sattam Bin Abdulaziz University, Al-Kharj, Saudi Arabia; ^6^Department of Zoology and Entomology, Faculty of Science, Helwan University, Cairo, Egypt; ^7^Department of Zoonoses, Faculty of Veterinary Medicine, Sohag University, Sohag, Egypt

**Keywords:** antiviral activity, propolis, chitosan nanoparticle, NDV, VII

## Abstract

The Newcastle disease virus (NDV) is considered a serious threat to global poultry production. Despite the availability of vaccines, it remains a major devastating epidemic responsible for great economic losses. The development of novel virus-controlling strategies is therefore an urgent need. The present study investigated for the first time the antiviral efficacy of propolis and chitosan nanoparticles against two NDV isolates, MW881875 and MW881876, recovered from vaccinated commercial broiler farms in KafrEl Sheikh Governorate, Egypt. The polygenetic analysis focused on the *F* and *M* genes, with one isolate having a 97% identity with the genotype VII NDV Israeli strain. On the other hand, the identified isolates showed high genetic variation and only 76% identity with the LaSota vaccine (genotype II). More interestingly, the cell cytotoxic concentrations of chitosan, propolis, and a propolis–chitosan mixture against Vero cells were 327.41 ± 12.63, 109.48 ± 8.36, and 231.78 ± 11.46 μg/ml, respectively. The median tissue culture infectious dose (TCID50) assay demonstrated that the nanoparticles have antiviral effects after NDV exposure resulting in significant decrease in viral titer (TCID50) by 2, 2.66, and 2.5 log10 at 62 μg/ml of chitosan, 13 μg/ml of propolis, and 30 μg/ml of the propolis–chitosan mixture, respectively, compared with the control TCID50 value of 4 log10. Taken together, the results provide novel insights into the potentially promising roles of propolis and chitosan as novel, safe, and effective antiviral agents against NDV.

## Introduction

Newcastle disease (ND) remains one of the most serious viral diseases affecting poultry farms in many developing countries including Egypt (1–3). Although vaccines have been used to control ND in most broiler farms for more than 60 years, ND remains the most dangerous infectious poultry disease worldwide (4). ND is caused by the ND virus (NDV), an avian paramyxovirus serotype 1 that can infect more than 200 different bird species. It is an enveloped negative-sense RNA virus with a single-stranded RNA genome from the genus Avulavirus and the family Paramyxoviridae (5). The NDV is divided into two classes; class I consists of one main genotype (mainly avirulent strains), while class II is of low, moderate, and high virulence, and contains 20 genotypes (I–XXI), including genotype XV that contains only recombinant sequences and is excluded from the final analyses (6). The envelope has two surface glycoproteins, hemagglutinin–neuraminidase, which is important for virus attachment to host cell membrane, and the fusion protein, which is responsible for virus fusion with the host cell membrane (7). Because they are considered immune response targets, the fusion and hemagglutinin–neuraminidase proteins guard against infection with virulent NDV strains (8). The emergence and spread of new genotypes around the world contribute to the continuous evolution of velogenic ND strains, leading to more variation, and is considered a major threat to poultry (9, 10). The available antivirals have many side effects such as toxicity and emergence of drug-resistant strains. Clearly, there is an urgent need for developing safe and effective antiviral agents.

Interestingly, nanoparticles have been found to be effective against a wide range of pathogens, including bacteria, fungi, parasites, and viruses (11). It is worth noting that nanomaterials have numerous physicochemical properties, including nanosize, high area-to-mass ratio, and high reactivity. Nanoparticles can occur naturally or be intentionally designed (12), and they have a potential as novel and potent antiviral agents to overcome the limitations of conventional therapeutic agents (13). Furthermore, nanoparticles have the ability to control important functions such as blood circulation half-life, drug-release characteristics, immunogenicity, solubility, and diffusivity. Chitosan is a natural polymer produced by alkaline hydrolysis of chitin or fungal strain fermentation. Chitin can be found in exoskeletons of arthropods, crustacean shells, and insect cuticles. Importantly, chitosan is a good candidate for encapsulation as it can be combined with polymers, metals, and ceramic materials to form composites, in addition to having a wide range of biological properties including non-toxicity, biocompatibility, and biodegradability, as well as antibacterial (14), antimicrobial, antiviral, and fungicidal properties (15, 16). It can also be used as a drug carrier in a variety of ways. The interaction of cationic chitosan with an anionic cell surface is thought to be the mechanism of action against bacteria, but it may also prevent protein synthesis by interfering with mRNA synthesis (17).

Propolis, also known as bee putty or bee glue, is a natural plant product found in beehives and collected and treated by bees (18). A literature review of several reports documented the antimicrobial effects of propolis related to the presence of flavonoids and terpenoids (19). Among others, propolis flavone is a vital constituent of propolis that is used as an adjuvant and antiviral agent in chickens injected with activated or inactivated vaccines. Propolis flavone has also been shown to improve the immune-enhancing activity in both cellular and humoral immune responses (20). A previous study reported a successful preparation of live vaccines against the NDV encapsulated in chitosan nanoparticles (21). However, no previous studies have investigated the antiviral activity of propolis and chitosan nanoparticles against genotype VII NDV. Therefore, the present study conducted an *in vitro* assay to assess the potential influence of propolis and chitosan nanoparticles on NDV infectivity.

## Materials and methods

### Ethical consideration

The study was approved by the Research, Publication, and Ethics Committee of the Faculty of Veterinary Medicine, Kafrelsheikh University, Egypt, with Institutional Review Board number KFS-2019/6.

### Sampling

A total of 600 samples (200 lungs, 200 cecal tonsils, and 200 tracheae) were routinely collected from both apparently healthy and morbid broiler chickens aged 15–29 days from 60 poultry farms (broiler) in Kafr El Sheikh Governorate, Egypt between October 2019 and October 2020. Each bird's lung, cecal tonsils, and trachea were pooled together to create 200 working samples. The samples were collected, handled, preserved, and analyzed in the laboratory in accordance with the World Health Organization's recommendations. The samples were ground in phosphate-buffered saline containing 1,000 IU/ml of penicillin G-sodium and 1 mg/ml of streptomycin sulfate. The samples were iced for three cycles, liquefied, and centrifuged at 4,032 × g for 5 min to collect supernatants, which were then stored at −80°C until use.

### Virus isolation

The prepared samples were inoculated into the allantoic cavity of 10-day-old specific pathogen-free embryonated chicken eggs (five eggs for each sample). For 3 days, the eggs were incubated at 37°C and candled daily (22). Slide hemagglutination tests were performed on the collected allantoic fluid.

### RNA extraction

Total RNA was extracted from the prepared samples (23) using a QIAamp Viral RNA Mini kit according to the manufacturer's instructions.

### Reverse transcriptase-polymerase chain reaction

The extracted RNA was tested for NDV using a One-step RT-PCR kit (Qiagen, Hilden, Germany) according to the manufacturer's instructions. The cDNA was then used to amplify partial NVD *M* and *F* gene sequences using a gene-specific forward primer M2 (5′ TGG-AGC-CAA-ACC-CGC-ACC-TGC-GG 3′) and a reverse primer F2 (5′ GGA-GGA-TGT-TGG-CAG-CAT-T3′). PCR cycling was performed on a T3 Biometra thermocycler (Analytik Jena GmbH, Jena, Germany), with the following PCR thermal profile: RT reaction at 50°C for 30 min, initial PCR activation at 95°C for 5 min, 36 three-step cycles of denaturation at 94°C for 30 s, annealing at 50°C for 45 s, extension at 72°C for 45 s, and final extension at 72°C for 10 min (24). For this step, LaSota Live Vaccine was kindly provided by Veterinary Serum and Vaccine Research Institute Abbasia (Cairo, Egypt) and served as a positive control while the transport medium was used as a negative control.

### Sequencing and phylogenetic analysis

The amplified DNA bands of NDV PCR products for the *F* gene (partial F gene sequence) were excised from agarose gels, purified with a Montage DNA Gel Extraction kit (Millipore, Burlington, MA, United States), and sequenced on an automated ABI 3730 DNA sequencer (Applied Biosystems, Foster City, CA, United States). The sequences were aligned using the Clustal W tool. The nucleotide sequences were matched with sequences from the National Center for Biotechnology Information GenBank using the MEGA V5.20 software. Bootstrap values were used for 1,000 alignment replicates.

### Propolis extract preparation

Propolis was obtained from the Animal Health Research Institute in Egypt and finely ground. Later, a 25-g sample of propolis was liquefied in 250 ml of ethanol (80% v/v) for 1 day at room temperature using a magnetic mixer. The propolis extract was then filtered and centrifuged at 8,673 × g for 30 min to produce ethanolic propolis extract (EEP). The samples were stored at room temperature and in the dark until further use (25).

### Chitosan nanoparticle preparation

Chitosan nanoparticles were prepared as described by Cortés-Higareda et al. (26). In brief, 0.1 g of chitosan was mixed with 1% (v/v) glacial acetic acid to make a 0.1% (w/v) chitosan solution. At room temperature, 1 ml of tripolyphosphate solution was added to 25 ml of chitosan solution on a magnetic stirrer. After stirring for 20 min, the mixture was placed in a rotary evaporator at 40°C to evaporate the solvent. The resulting nanoparticles were refrigerated at 4°C.

### Propolis–Chitosan mixture preparation

Before lyophilizing or drying, an aliquot of 2.5 ml of the chitosan solution's aqueous phase was added to 40 ml of the organic phase (ethanolic propolis extract) with 10 μl of Tween 20 using a peristaltic pump and with constant magnetic stirring. The solution was then placed in a rotary evaporator at 40°C to evaporate the solvent, and the resulting nanoparticles were refrigerated at 4°C.

### Transmission electron microscopy

The nanoparticles' size and shape were measured using a JEOL JEM-1010 microscope (JEOL, Peabody, MA, United States) with a voltage of 60 kV. The average size was calculated using the ImageJ software. To prepare for analysis, one drop of the sample was placed on a copper grid (27).

### Mammalian cell culture and virus propagation

Vero cells derived from kidneys of African green monkeys were obtained from the American Type Culture Collection (Manassas, VA, United States). The cells were prepared as previously described (28). The cytopathogenic NDV was then propagated and assayed in confluent Vero cells (29). The infectious NDV isolates named MW881875 and MW881876 were counted using the Spearman–Karber method by determining the 50 percent tissue culture infective dose (TCID50) with eight wells per dilution and 20 μl of inoculum per well (30).

### Cytotoxicity assay

The Vero cell lines were seeded on 96-well plates at a density of 2 × 10^5^ cells/ml in 100 μl of a growth medium. After 24 h, a fresh medium with varying concentrations of the tested samples was added. Serial two-fold dilutions of the tested compounds (chitosan-propolis nanoparticles mixture) ranging from 2 to 3,000 μg/ml dissolved in 0.1% dimethyl sulfoxide (DMSO) were dispensed onto 96-well flat-bottomed micro titer plates (Falcon, Jersey, NJ, United States) containing confluent cell monolayers using a multichannel pipette. DMSO at as concentration of 0.1% is known to have no inhibitory effects on Vero cell growth (31). The microtiter plates were then incubated for 48 h at 37°C in a humidified incubator with 5% CO_2_. Each concentration was placed in three wells of the sample of interest. After incubation, viability was determined using the 3-(4,5-dimethylthiazol-2-yl)-2,5-diphenyl-2H-tetrazolium bromide (MTT) colorimetric assay as previously described (32). The assay also included wells composed of 100 μl of growth media without Vero cells that served as negative control. Meanwhile, positive control wells composed of Vero cells incubated with 0.1% of DMSO and neither infected with NDV nor treated with any nanocompound were also included.

### Evaluation of antiviral activity

The antiviral effects were assessed using a cytopathic inhibition assay at the Regional Center for Mycology and Biotechnology, Al-Azhar University, Cairo, Egypt. Using the MTT method, the cytopathic effect of compounds on mammalian cells in tissue culture was determined (33). In brief, a Vero cell monolayer of 2 × 10^5^ cells/ml adhered to the bottom of a 96-well microtiter plate was incubated for 24 h at 37°C in a humidified incubator with 5% CO_2_. The plates were washed with fresh Dulbecco Minimum Essential Medium and tested with a 10^4^ TCID50 dose of the virus before being treated with all the test nanoparticles independently in a fresh maintenance medium after 1 h. The cells were then incubated at 37°C for 48 h. Infection controls composed of Vero cells neither infected with NDV nor treated with any nanocompound served as negative control, and Vero cells infected only with the NDV but not treated with any of the test compounds were used as positive control. Each concentration of each test compound was tested in six wells. The cytopathic effects were compared to the controls to assess the antiviral activity and measure the protective effect of each test compound test on the cells. Three separate experiments were performed, each with four replicates per treatment. Amantadine was used as a reference drug in this assay as described elsewhere (34). The viability of cells after incubation was determined using the MTT assay as described previously. Viral inhibition rate was calculated as follows: $\left[\frac{A-B}{C-B}\right]\times \;100\%,$ where A, B, and C represent the absorbance of the test compounds in virus-infected cells, virus control absorbance, and cell control absorbance, respectively.

### Virus titration

The virus infection titer of the NDV isolates named MW881875 and MW881876 was estimated by serially diluting the cell supernatant 10 times. In brief, 200 μl of the cell supernatant was added into a test tube, followed by 1.8 ml of serum-free fresh media. The solution was thoroughly mixed with a pipette, and 200 μl of the mixture was transferred from the first tube to the second tube, which contained 1.8 ml of fresh media, and homogenized thoroughly by pipetting. This process was repeated more than five times for a total of seven dilutions (10^1^-10^8^). The diluted viruses were added to a 96-well plate of precultured Vero cells plates and then covered with a sterilized sealing film. The viral cytopathic effect was examined using an inverted microscope as described by Ramakrishnan (35). Infectivity titer was calculated as follows:


$$\begin{array}{l} PD\;=\;\%CPE\;>\;50\%-50\%\;const\;/\;\%CPE\;>\;50\%\\ \;-\;\%CPE\;<\;\%\times log \end{array}$$

### Data analysis

Half maximal effective concentration (EC_50_), which is defined as the dose that prevents viral infection in 50% of cells, was calculated from the obtained data using the Stata modeling software (StataCorp. 2021. Stata Statistical Software: Release 17. College Station, TX: StataCorp LLC). Selectivity index (SI) was calculated as the ratio of 50% cytotoxic concentration (CC_50_) to EC_50_ (33).

## Results

### Virus isolation and identification

In the present study, stunted growth, congestion, and embryonic death were observed on the third egg passage 72 h post infection as compared to non-infected control eggs. More importantly, the conventional RT-PCR for NDV detection by amplification of the *M* and *F* gene parietal sequences revealed that 140 of the 200 samples tested were positive, with a specific band at 766 pb. The results of virus isolation from the 60 broiler farms in Kafrelsheikh are shown in Supplementary Table 1.

### Phylogenetic analysis

**Figure 1:**
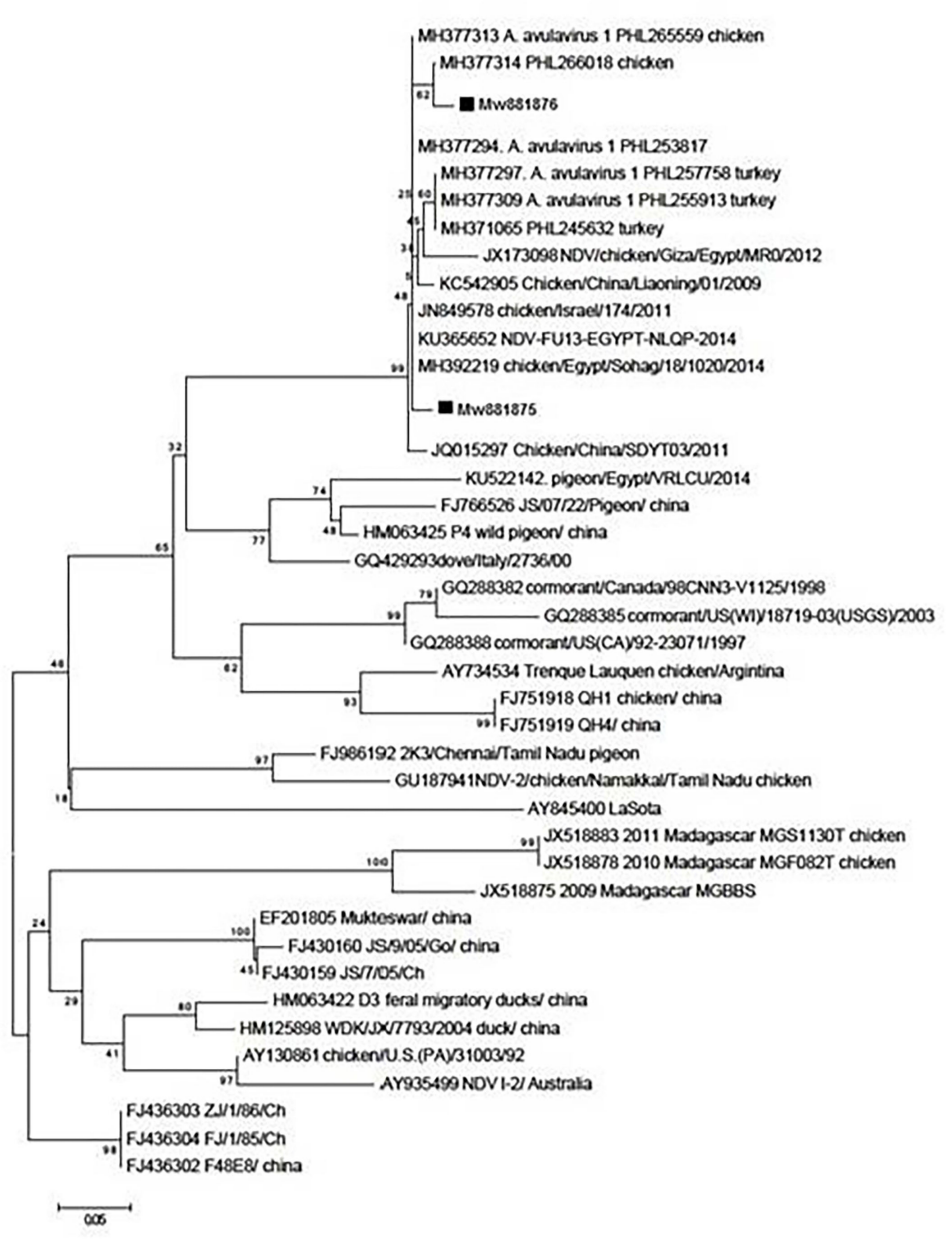
Phylogenetic analysis of nucleotide sequences of the partial fusion genes *F* and *M* from two Egyptian samples with other sequences of the reference strains from GenBank using the neighbor-joining method and 1,000 bootstrap replicates.

A phylogenetic tree was constructed using a sequence of the *F* gene end and the *M* gene beginning (Figure 1). The nucleotide sequences of the examined isolates were named MW881875 and MW881876. The sequences clustered in genotype VII of the current study revealed 97.66% genetic similarity to Israeli strains (MH377313–MH377294) and 97.52, 97.38, and 97.25% similarity to the Egyptian strains Luxor (MK495897), Sadat Menoufia (MG717686), and Sohag (MK673139), respectively. There was also a limited genetic similarity of 76% to the genotype II LaSota vaccine.

### Characterization of nanoparticles

**Figure 2:**
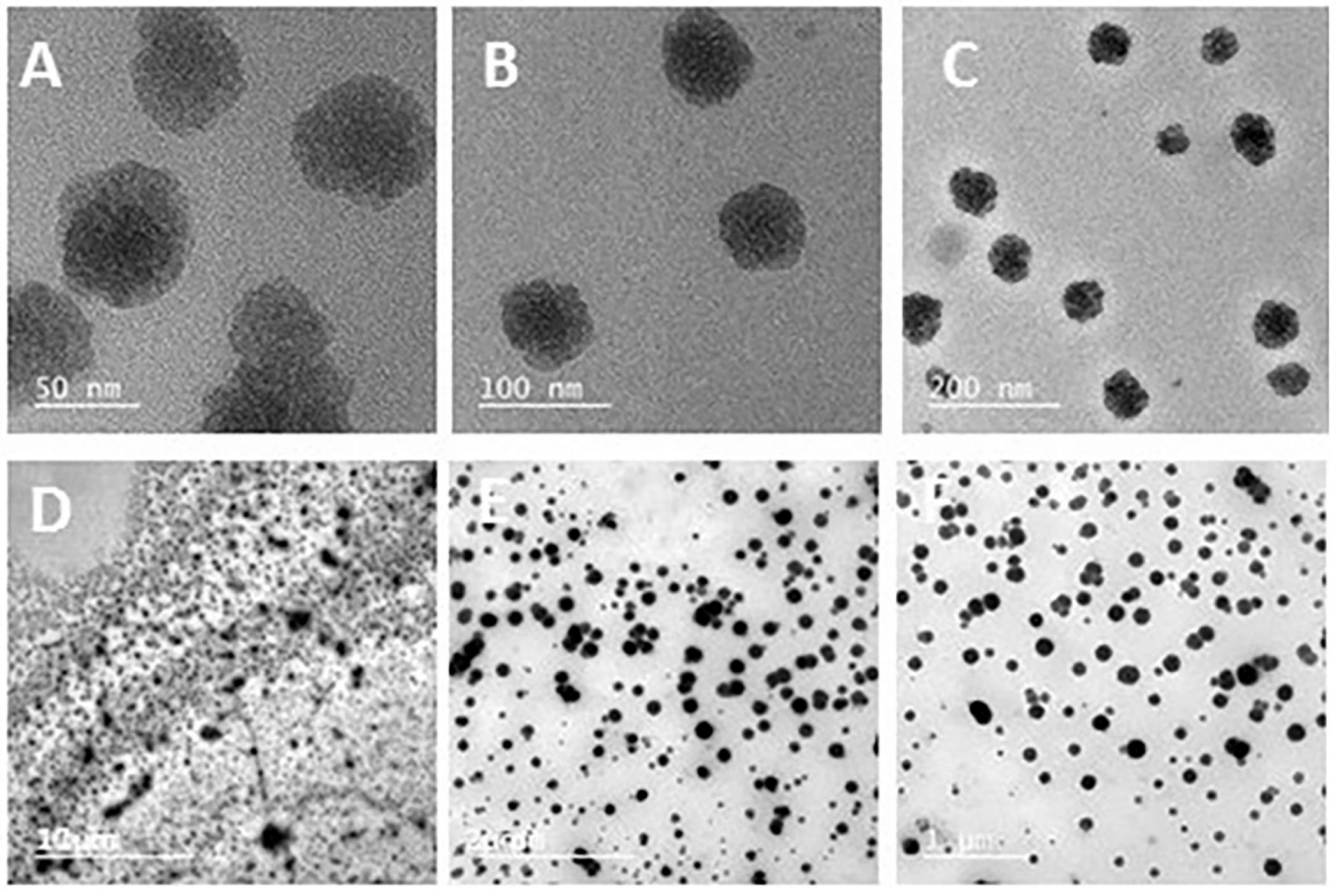
Transmission electron microscopy of chitosan nanoparticles showing spherical shapes with uneven borders and uniformity, with average diameters ranging from 50 to 200 nm **(A–C)**, as well as a propolis–chitosan mixture **(D–F)**.

In the present study, chitosan nanoparticles spherical in shape and with uneven borders were dispersed. As shown in Figure 2, the average diameter of chitosan ranged from 50 to 200 nm. The chitosan particles were 0.5–2 mm in size, and when the propolis extract was added, they formed round aggregations.

### Cytotoxicity assays

**Figure 3:**
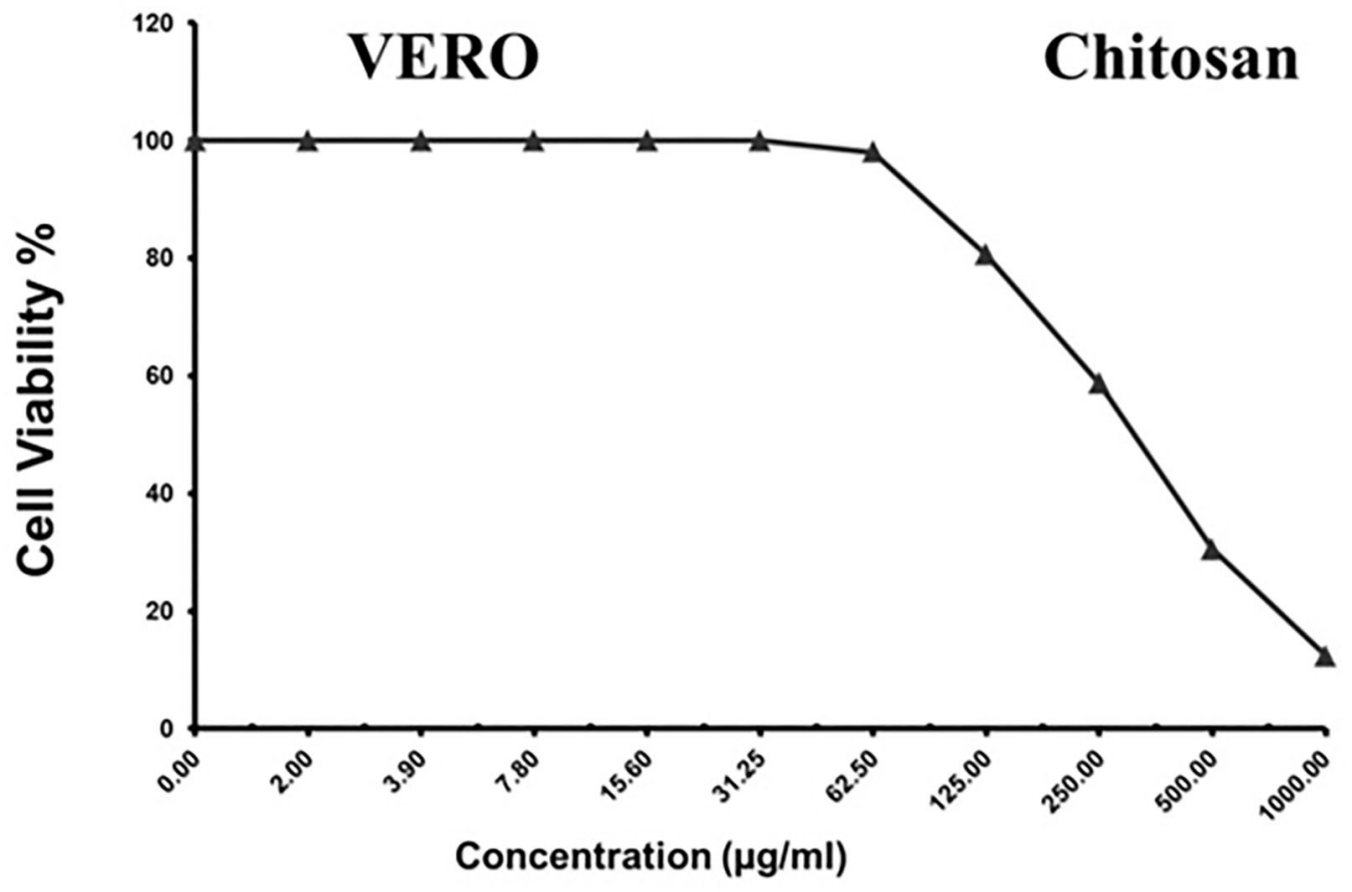
Cytotoxic activity of chitosan in mammalian cells derived from African Green Monkey kidney (Vero) cells at a 50% cell cytotoxic concentration.

**Figure 4:**
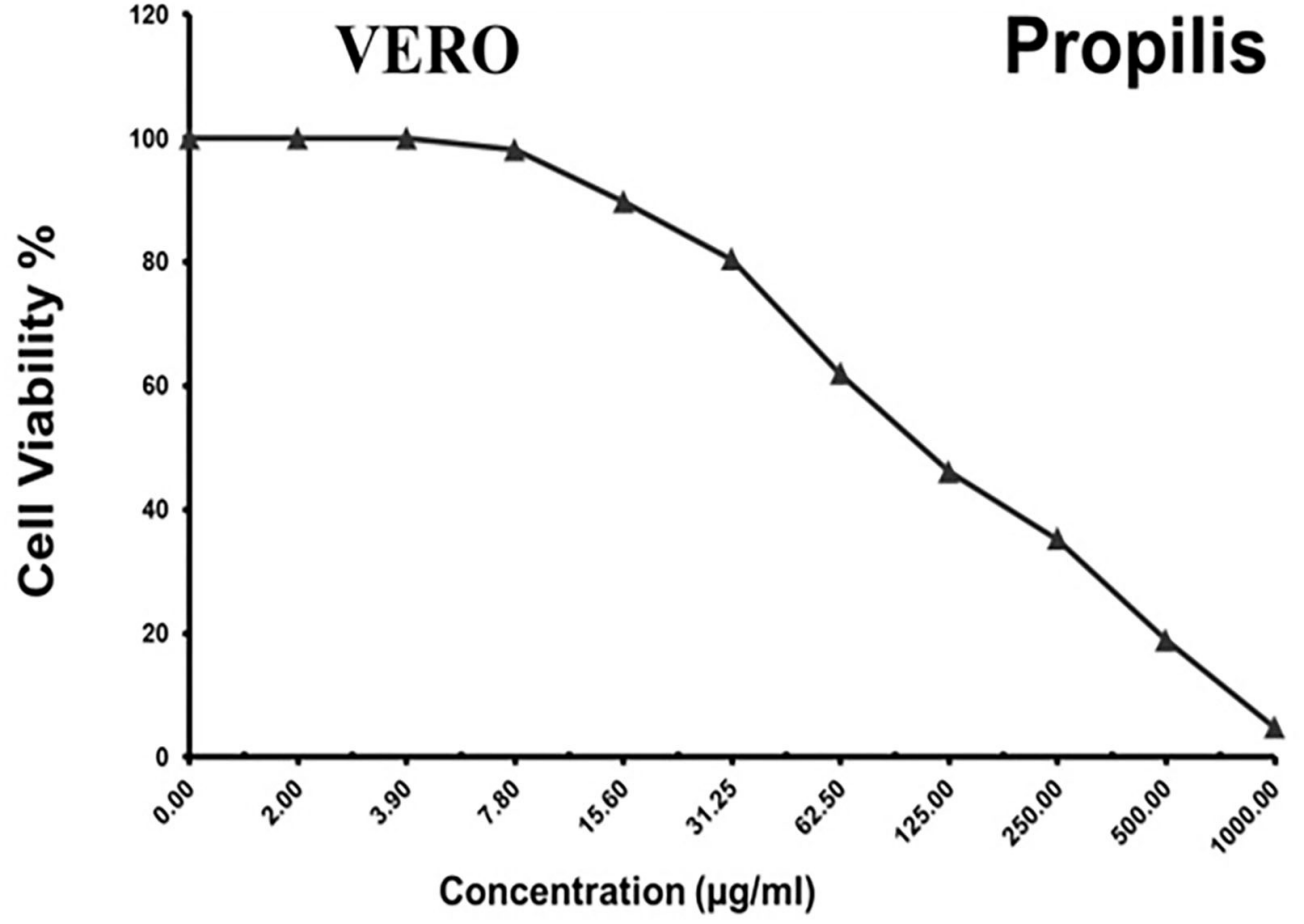
Cytotoxic activity of propolis in mammalian cells derived from African Green Monkey kidney (Vero) cells at a 50% cell cytotoxic concentration (CC_50_).

**Figure 5:**
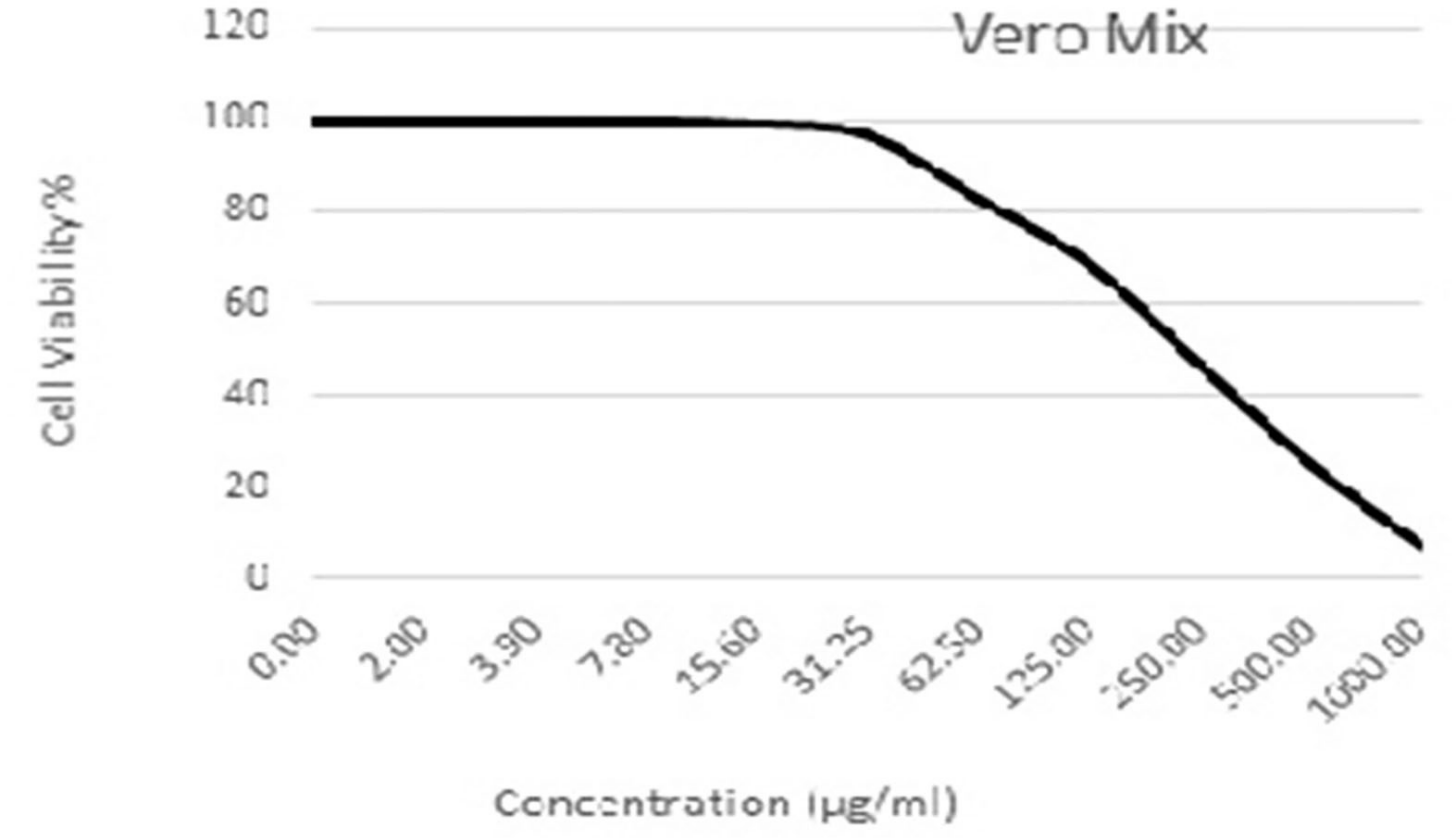
Cytotoxic activity of the propolis–chitosan mixture in mammalian cells derived from African Green Monkey kidney (Vero) cells at a 50% cell cytotoxic concentration (CC_50_).

As shown in Figures 3–5 and Tables 1–3, the cytotoxic activity of chitosan, propolis, and the propolis–chitosan mixture against Vero cells was measured, and their CC_50_ was 327.41 ± 12.63, 109.48 ± 8.36, and 231.78 ± 11.46 μg/ml, respectively. However, the cell viability exceeded 90% when 62, 13, and 30 μg/ml of chitosan, propolis, and the mixture, respectively, were added.

**Table 1:** Cytotoxic activity of chitosan in mammalian cells derived from African Green Monkey kidney (Vero) cells at a 50% cell cytotoxic concentration.

**Sample conc**.	**Viability**	**Inhibition**	**SD**
**(μg/mL)**	**(%)**	**(%)**	**(±)**
1,000	12.34	87.66	1.28
500	30.47	69.53	2.91
250	58.76	41.24	3.46
125	80.53	19.47	1.23
62.5	98.06	1.94	0.32
31.25	100	0	
15.6	100	0	
7.8	100	0	
3.9	100	0	
2	100	0	
0	100	0	0

**Table 2:** Cytotoxic activity of propolis in mammalian cells derived from African Green Monkey kidney (Vero) cells at a 50% cell cytotoxic concentration.

**Sample conc**.	**Viability**	**Inhibition**	**SD**
**(μg/mL)**	**(%)**	**(%)**	**(±)**
1,000	4.76	95.24	0.62
500	18.83	81.17	0.95
250	35.19	64.81	1.37
125	46.08	53.92	2.84
62.5	61.87	38.13	3.15
31.25	80.42	19.58	1.86
15.6	89.75	10.25	0.91
7.8	98.13	1.87	0.65
3.9	100	0	0
2	100	0	0
0	100	0	0

**Table 3:** Cytotoxic activity of a chitosan-propolis nanoparticle mixture in mammalian cells derived from African Green Monkey kidney (Vero) cells at a 50% cell cytotoxic concentration.

**Sample conc**.	**Viability**	**Inhibition**	**SD**
**(μg/mL)**	**(%)**	**(%)**	**(±)**
1,000	7.29	92.71	1.19
500	25.13	74.87	2.63
250	46.80	53.2	2.25
125	68.74	31.26	4.29
62.5	82.95	17.05	2.74
31.25	96.71	3.29	1.22
15.6	99.23	0.77	0.79
7.8	100	0	
3.9	100	0	
2	100	0	
0	100	0	

### Assessment of antiviral activity

As presented in Table 4, the inhibition ratios of chitosan, propolis, and the mixture at the minimum non-cytotoxic concentration (MNCC) of 62, 13, and 30 μg/ml resulted in prevention rates of 25, 2.6, and 37%, respectively, based on NDV viral loads. The anti-NDV activity of the mixture was greater than that of chitosan or propolis alone, and NDV production was inhibited by amantadine at a dose of 150 μg/ml, with a reduction rate of 71.84%.

**Table 4:** Antiviral effects of the test compounds against Newcastle disease virus at maximum non-cytotoxic concentration (MNCC).

**Sample name**	**MNCC (μg/mL)**	**Antiviral effect on**	**Viral titers**	**Antiviral efficiency**		
		**NDV virus (%)**				
			**Log 10**	**EC_50_**	**CC_50_**	**SI**
Propolis	13	25.48 ± 3.56	2.66	78.31 ± 5.17	109.48 ± 8.36	1.39
Chitosan	62	2.64 ± 0.72	2	579.42 ± 32.54	327.41 ± 12.63	0.55 (inactive)
Propolis andchitosan mixture	30	37.16 ± 3.88	2.5	148.26 ± 23.78	231.78 ± 11.46	1.56
Amantadine (reference drug)	150	71.84 ± 4.28	1	39.86 ± 3.42	354.93 ± 61.85	8.9

### Virus titration

**Figure 6:**
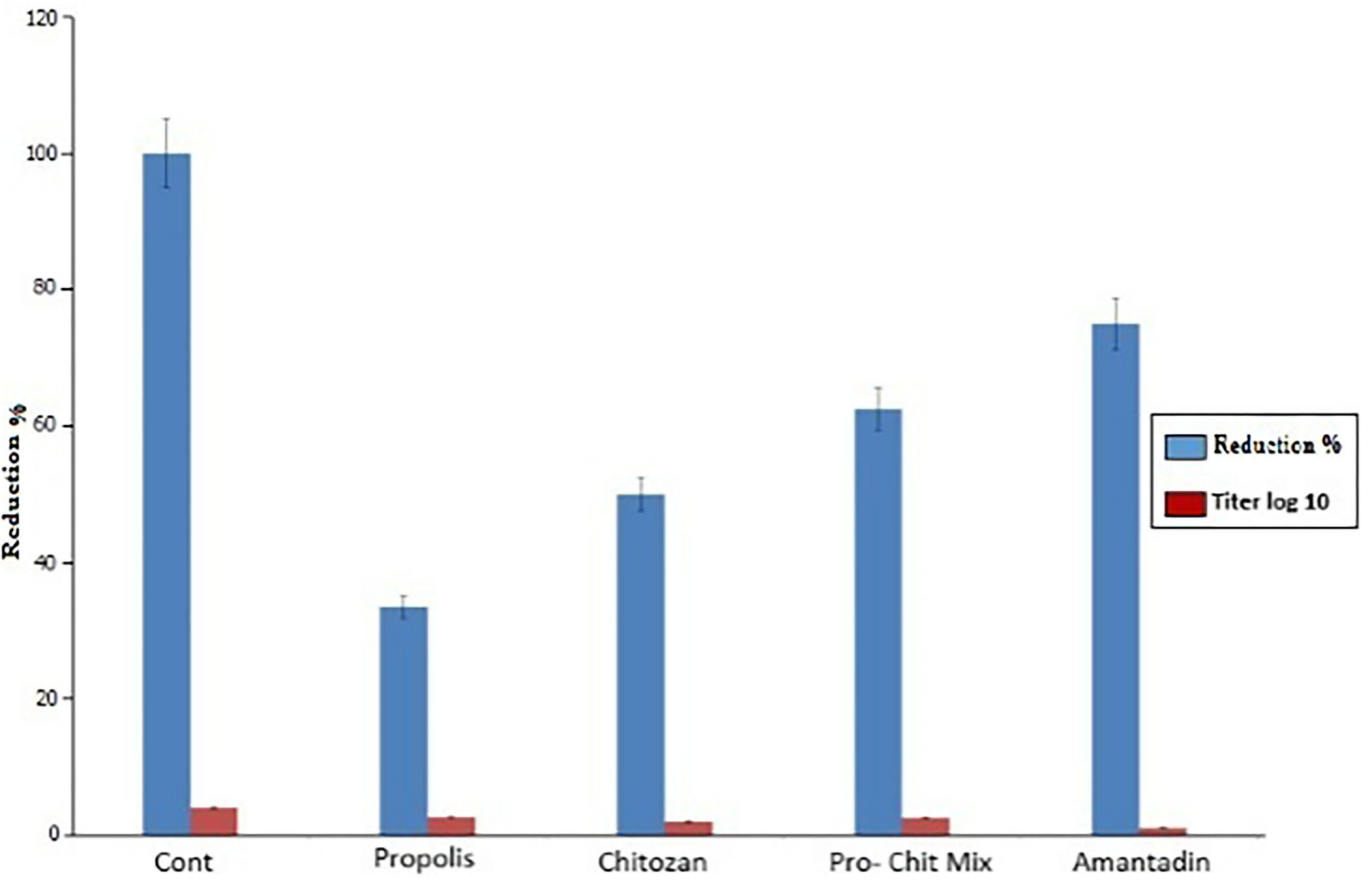
Assessment of antiviral activity of propolis, chitosan, the propolis–chitosan mixture, and amantadine on the Newcastle disease virus titer by 50% tissue culture infectious dose (TCID50) assay (*p* < 0.0001).

In this study, the virus titration using TCID50 assays revealed that chitosan, propolis, and the propolis–chitosan mixture of nanoparticles had antiviral effects after NDV exposure, with a significant decrease in viral titer. As shown in Figure 6, viral concentrations of 62, 13, and 30 μg/ml resulted in TCID50 reductions of 2, 2.66, and 2.5 log10, respectively, when compared to the control TCID50 value of 4 log10.

## Discussion

ND remains one of the most serious avian respiratory diseases affecting the majority of poultry farms in several countries and causing severe economic losses (1–3). For several years, genotype VII NDV has been the most widely circulating virus in various regions, including South Africa, China, Europe, and the Middle East (36, 37). To date, ND vaccines have failed to provide complete protection for poultry against new genotype VII isolates. A previous study revealed that chickens vaccinated with LaSota vaccine's variant VII developed clinical symptoms, indicating that the genotype VII virus can cause disease in vaccinated birds (38, 39). This is due to the large antigenic and phylogenetic distances between the vaccines and recently circulating virulent NDV strains, which may facilitate the development of virulent NDV (40). The use of genotype-matched vaccines may significantly reduce viral shedding (41), but it does not provide complete protection. In Egypt, to control the NDV, routine vaccine programs should clearly match recently isolated and genotyped field strains. Furthermore, developing an effective molecular diagnostic strategy with novel drug targets may aid in disease control.

It is worth noting that RT-PCR is a more sensitive and rapid method for detecting and differentiating NDV subtypes than conventional methods (42, 43). Molecular typing and phylogenetic analysis of the *F* gene are considered a major NDV determinant (44, 45). In the present study, RT-PCR was conducted to identify the isolates, and 140 of the 200 tested pooled samples were positive, with an amplicon size of 766 bp. The molecular analysis identified two NDV isolates (MW881875 and MW881876) recovered from vaccinated commercial broiler farms in KafrEl Sheikh Governorate, Egypt. Molecular identification was followed by polygenetic analysis of the recovered isolates by targeting the *F* and *M* genes, with the recovered one having 97% identity with the genotype VII NDV Israeli strain, while the other one had a high genetic variation and only 76% identity with the LaSota vaccine (genotype II). The current findings are consistent with a previous study (46), which identified isolates related to genotype VII NDV Chinese strains based on F protein sequence analysis. Furthermore, it has been reported that the sub-genotype VII.1.1 is most prevalent in Egypt and is responsible for multiple NDV outbreaks in poultry (1–3, 47).

It is worth noting that the current antiviral drugs have significant adverse effects, including the emergence of resistant strains during treatment, in addition to their use at high concentrations (48). On the other hand, nanomaterials are a cost-effective technology that has been used for several decades to treat a variety of pathological conditions (49–52). Among others, nanoparticles have various effects against viruses, including drug-resistant viruses, with different types of coating, and nanoparticle synthesis is less expensive than conventional treatments (50). Importantly, the present study provided novel baseline data on the potential benefits of using propolis and chitosan nanoparticles together against molecularly identified isolates. In this study, the cytotoxic activity of propolis, chitosan, and a propolis–chitosan mixture against mammalian Vero cells was observed. We observed that propolis, chitosan, and propolis–chitosan mixture concentrations of 13, 62, and 30 mg/ml resulted in viability rates of 98.13, 98.06, and 99.23%, respectively, indicating that low doses of nanoparticles induced high viability of tissue culture cells. Interestingly, we found that combining propolis and chitosan resulted in a reduction (antiviral effect) of 37.16% at a maximum non-cytotoxic concentration of 30 mg/ml, with an SI of 1.56, which is much better than using either compound alone.

Propolis is a natural plant product produced by *Apis mellifera* bees (18). Interestingly, it contains over 300 biochemical constituents including flavonoids, polyphenols, phenolic aldehydes, sesquiterpenes, and coumarins rendering it a potent antimicrobial activity (53–55). Several previous reports have shown that propolis has an antiviral activity against the *Vaccinia* virus, *Herpes Simplex* virus, *retroviruses*, influenza viruses, and, most recently, severe acute respiratory syndrome–related coronavirus (SARS-CoV-2) (54, 56, 57). Taken into account, the presence of flavonoids, caffeic acid, and esters of aromatic acids is mostly responsible for the antiviral activity of propolis through their role in preventing virus transmission to other cells, inhibiting virus propagation, and abolishing the virus's external envelope (58). Chitosan is a natural component with potent antibacterial activity (59). The antibacterial properties of chitosan can be explained by its positively charged amine groups, which can interact with the negatively charged bacterial cell membrane and thus may bind to DNA, inhibiting mRNA and protein synthesis (60). Importantly, the use of nanoparticles for therapeutic purposes depends on their safety in host tissues, which can be assessed by *in vitro* cytotoxicity assay. It should be noted that compounds with an SI of 2 or higher are considered active (33), since it means that their antiviral activity is sufficiently higher than their toxicity. Several previous reports (61) found that adding nanoparticles to edible coatings improved their properties, and that combining chitosan and propolis enhanced their antimicrobial potential.

On the other hand, no antiviral activity was detected for chitosan alone in the present study since the administration of 62 mg/ml only resulted in 2.64% reduction in viral titer and an SI below 1 (0–0.55), indicating its inactivity. These findings are consistent with those of a previous study (62), which found that chitosan alone has no antiviral activity, but that a combination of silver nanoparticles and chitosan has, implying that silver nanoparticles are required to exert the antiviral effect of the combination. However, mechanisms underlying composite antiviral activity remain unknown. Meanwhile, propolis alone reduced cytopathic effects by 25.48% at 13 MNCC doses, with an SI of 1.39. In the present study, the addition of silver nanoparticles 1 h after infection reduced NDV proliferation because of the inhibitory activities of the nanoparticles. A previous study (63) found that treatment of MDCK-SIAT1 cells with nanoparticles prior to infection, as well as co-exposure of cells to nanoparticles with infection, did not reduce the H1N1 influenza virus titer. These findings indicate that the nanoparticles only exert their effects after the viruses entered the cells, not before or during the attachment of influenza virus particles to host cells.

## Conclusions

Taken together, the present study revealed that genotype VII is the main isolate obtained from vaccinated broiler farms. Furthermore, the detected isolates (MW881875 and MW881876) shared a close similarity to Israeli, Egyptian, and Chinese strains, but differed from LaSota vaccine strains. More importantly, our *in vitro* study investigated the potential protective effects of propolis, chitosan, and their combination against the isolated genotype VII of the NDV. Interestingly, while the propolis–chitosan mixture demonstrated a promising antiviral activity, chitosan alone had no antiviral effect. Meanwhile, propolis alone demonstrated a moderate antiviral activity with low cytotoxicity to tissue culture cells, indicating that propolis may be an effective new antiviral agent against NDV infection. Further future studies are required to elucidate the exact antiviral mechanism of these nanoparticles. Furthermore, future *in-vivo* studies using propolis and chitosan nanoparticles to control Newcastle disease (ND) infection genotype VII.1.1 (velogenic pathotype) should be conducted.

## Data availability statement

The original contributions presented in the study are included in the article/Supplementary material, further inquiries can be directed to the corresponding author/s.

## Ethics statement

The animal study was reviewed and approved by the guidance of Research, Publication, and Ethics of the Faculty of Veterinary, Kafrelsheikh University, Egypt, and the institutional Review Board Number KFS-2019/6.

## Author contributions

NA, SK, AT, HS, and AM designed the concept, performed the methodology, formal analysis, data curation and supervision, and revised the manuscript. HG, ML, and EE participated in the methodology, formal analysis, data curation, and scientific advice. NA, SK, AT, HS, AM, and EE drafted the manuscript and prepared it for publication and revision. All authors have read and approved the final version of the manuscript.

## Conflict of interest

The authors declare that the research was conducted in the absence of any commercial or financial relationships that could be construed as a potential conflict of interest.

## Publisher's note

All claims expressed in this article are solely those of the authors and do not necessarily represent those of their affiliated organizations, or those of the publisher, the editors and the reviewers. Any product that may be evaluated in this article, or claim that may be made by its manufacturer, is not guaranteed or endorsed by the publisher.
