# Supplementary material for: In vitro investigation of the antiviral activity of propolis and chitosan nanoparticles against the genotype VII Newcastle disease virus
Source: Front Vet Sci. 2022 Aug 25;9:947641. doi: 10.3389/fvets.2022.947641 (PMC9453155; doi:10.3389/fvets.2022.947641)
Supplement: Supplementary file 1 [file Table_1.DOCX]

Title: *In vitro* investigation of the antiviral activity of propolis and chitosan nanoparticles against the Genotype VII Newcastle disease virus

| **PCR positive samples** | **Vaccination for ND** | **Mortality percent** | **Age (day)** | **Total n. of**  **pooled**  **samples** | **Location** |
| --- | --- | --- | --- | --- | --- |
| 30 | Live only Genotype II | 10 | 23 | 40 | Alhamoul |
| 23 | Inactivated +live attenuated | 15 | 20 | 40 | Desouk |
| 20 | Inactivated +live attenuated | 5 | 25 | 40 | Paltim |
| 35 | Inactivated +live attenuated | 20 | 29 | 40 | Kafrelsheikh |
| 32 | Live only Genotype II | 25 | 15 | 40 | Elreyad |

Noura Alkhalefa ^1^, Samy Khaliel ^2^, Abdelnaby Tahoon ^3^, Hanan Shaban ^3^, Asmaa Magouz ^1^, Hanaa Ghabban ^4^, Maha S. Lokman ^5,6^, and Ehab Kotb Elmahallawy *^7^

**Supplementary Table**

**Table S1**. Shows the results of virus isolation from 60 Broiler farms in Kafrelsheikh including [total No., age, mortality rate last 5 days of sampling, vaccination for ND (inactivated or live or both types of vaccines) indicated the genotype of the vaccine used] and results of PCR for each sample].
